# Heme molecule functions as an endogenous agonist of astrocyte TLR2 to contribute to secondary brain damage after intracerebral hemorrhage

**DOI:** 10.1186/s13041-017-0305-z

**Published:** 2017-06-24

**Authors:** Hyunjung Min, Boomin Choi, Yong Ho Jang, Ik-Hyun Cho, Sung Joong Lee

**Affiliations:** 1 Department of Neuroscience and Dental Research Institute, School of Dentistry, Seoul National University, 1 Gwanak-ro, Gwanak-gu, Seoul, 08826 Republic of Korea; 2 Department of Convergence Medical Science, College of Oriental Medicine, Kyung Hee University, Seoul, 02447 Korea

## Abstract

Toll-like receptor 2 (TLR2) was recently shown to contribute to secondary brain damage after intracerebral hemorrhage (ICH), although the molecular mechanisms of this contribution are elusive. In this study, we tested the hypothesis that hemin functions as a TLR2 endogenous agonist, causing proinflammatory astrocyte activation and secondary brain damage after ICH. Hemin administration to the mouse brain striatum induced ICH injury and neurological deficits, however, the brain injury volume and neurological deficits due to hemin injection were significantly reduced in TLR2 knock-out (KO) mice. Hemin administration induced neutrophil infiltration and upregulated neutrophil-attracting chemokine and proinflammatory cytokine expression in wild-type (WT) mice; these effects were ameliorated in TLR2 KO mice. Likewise, ICH-induced blood-brain barrier (BBB) damage was also decreased in TLR2 KO mice. This effect was most likely due to reduced matrix metalloproteinase 9 (MMP9) activity in the TLR2 KO mice compared to WT mice. In primary astrocytes, hemin directly induced MMP9 activity as well as proinflammatory cytokine and chemokine expression in a TLR2-dependent manner. Finally, hemin-induced MMP9 activity and proinflammatory gene expression were almost completely blocked by TLR2-neutralizing antibodies. Taken together, our data propose that heme released to the brain parenchyma after ICH injury activates TLR2 in astrocytes and induces inflammatory gene expression and BBB damage, which contribute to secondary brain damage after ICH.

## Background

Intracerebral hemorrhage (ICH) is one of the major types of stroke and accounts for 15% to 20% of all stroke cases. ICH begins with blood leakage into the brain parenchyma that causes brain damage, which is followed by inflammatory responses in the perihematomal area. Previous studies have indicated that inflammatory responses exacerbate ICH-induced injury. These inflammatory responses are accompanied by blood-brain barrier (BBB) disruption [1], glial cell activation, leukocyte infiltration, and induction of cytokine and chemokine expression, cumulatively resulting in hematoma expansion and neuronal damage [1, 2]. Hematomas and their degradation products have been suggested to trigger these inflammatory responses in the perihematomal region [3–5]. However, the molecular mechanisms underlying the inflammatory responses leading to secondary brain damage have not been completely elucidated.

In our previous study, we reported that Toll-like receptor 2 (TLR2), a pattern-recognition innate immune receptor, was required for inflammatory responses after collagenase-induced ICH in a mouse model [6]. TLR2 KO mice exhibited attenuated ICH-induced blood-brain barrier (BBB) damage, proinflammatory gene expression, and neutrophil infiltration compared to WT mice. Consequently, ICH-induced brain tissue damage and behavioral neurological deficits were reduced in TLR2 KO mice. In mechanistic studies, we demonstrated that astrocyte TLR2 activation increased matrix metalloproteinase 9 (MMP9) activity, which in turn compromised the BBB [6]. However, the precise molecule that activated astrocyte TLR2 during the initial phase of ICH damage, which triggered inflammatory responses and BBB damage, subsequently resulting in secondary brain damage, remained elusive.

It is well known that blood that has diffused into the brain parenchyma is highly toxic to brain tissue. Moreover, the majority of these harmful effects can be attributed to heme molecules released from erythrocytes in hematomas. Since the micro-environment of the brain parenchyma does not support erythrocyte survival, these cells are prone to lysis within hematomas, and heme molecules are subsequently released from the breakdown of hemoglobin. The released hemeprotein-free heme molecules can then cause oxidative damage and inflammation [7]. In support of this possibility, a previous study showed that hemin administration into the brain parenchyma resulted in increased brain damage, as measured by water content and inflammatory gene expression, in the perihematomal tissue. These effects recapitulate the key features of secondary brain damage after collagenase-induced ICH [8]. Considering the putative role of the heme molecule and the requirement of TLR2 in secondary brain damage after ICH, we hypothesized that the heme molecule may function as an endogenous agonist of astrocyte TLR2, thereby triggering inflammatory responses and compromising the BBB after ICH. In this study, we tested this hypothesis using TLR2 KO mice in an ICH model.

## Results

**Fig. 1:**
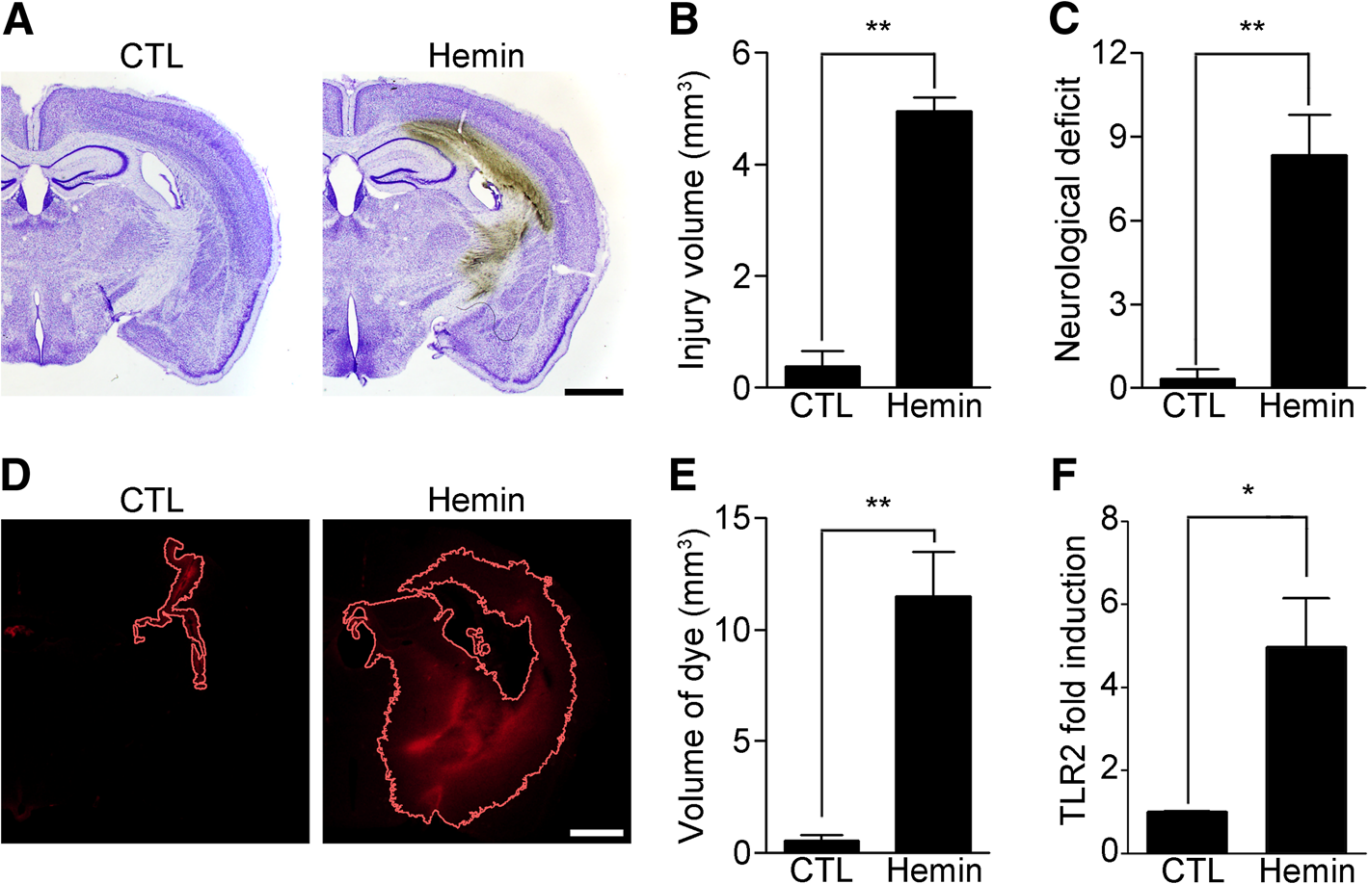
Hemin administration induces ICH injury. WT mice were administered 250 nmol of hemin in the striatum. **a** At 24 h after hemin injection, each brain was sectioned and used for cresyl violet staining to measure brain damage. Representative images are shown. Scale bar: 1 mm. **b** The injury volume (mm^3^) was calculated by multiplying the section thickness by the injured hemorrhagic area (** *p* < 0.01 vs. control mice, *n* = 5). **c** At 24 h following hemin injection, neurological deficits in the injured mice were evaluated using a 28-point neurological scoring system (** *p* < 0.01 vs. control mice). **d-e** BBB permeability after hemin injection was tested using Evans blue staining (D) from which the dye-stained volume was calculated (E) (** *p* < 0.01 vs. control mice, *n* = 4). **f** Total RNA was prepared from the injured brain hemispheres at 6 h after hemin injection (*n* = 3) and used for quantitative real-time RT-PCR to measure TLR2 mRNA levels (* *p* < 0.05 vs. control mice). For all graphs, the data are expressed as mean ± SEM

To test our hypothesis that the heme molecule functions as an endogenous agonist of TLR2 to induce neuroinflammatory responses during ICH, we first investigated if heme molecules in the brain parenchyma could induce pathological features comparable to those observed in collagenase-induced ICH [8]. To this end, we administered hemin, an oxidized heme molecule, into the striatum and measured the damaged tissue areas at 24 h after injection using cresyl-violet staining. In hemin-injected mouse brain tissue, injury was detected in the sub-cortical area near the needle injection site with a volume of 4.8 mm^3^. In contrast, no pronounced injury was detected in vehicle-injected mouse brain tissue (Fig. 1a, b). Collagenase-induced ICH results in neurological impairment and BBB damage [9]. Likewise, hemin-administered mice showed increased neurological deficit scores (Fig. 1c). In addition, hemin injection compromised the BBB near the injection site as assessed by Evans blue diffusion into the brain parenchyma (Fig. 1d). The dye-stained area around the injury site increased to 12 mm^3^ at 24 h after hemin injection compared to saline injection (Fig. 1d, e). These data show that direct hemin injection into the brain parenchyma recapitulates the pathological hallmarks of the collagenase-induced ICH model.

**Fig. 2:**
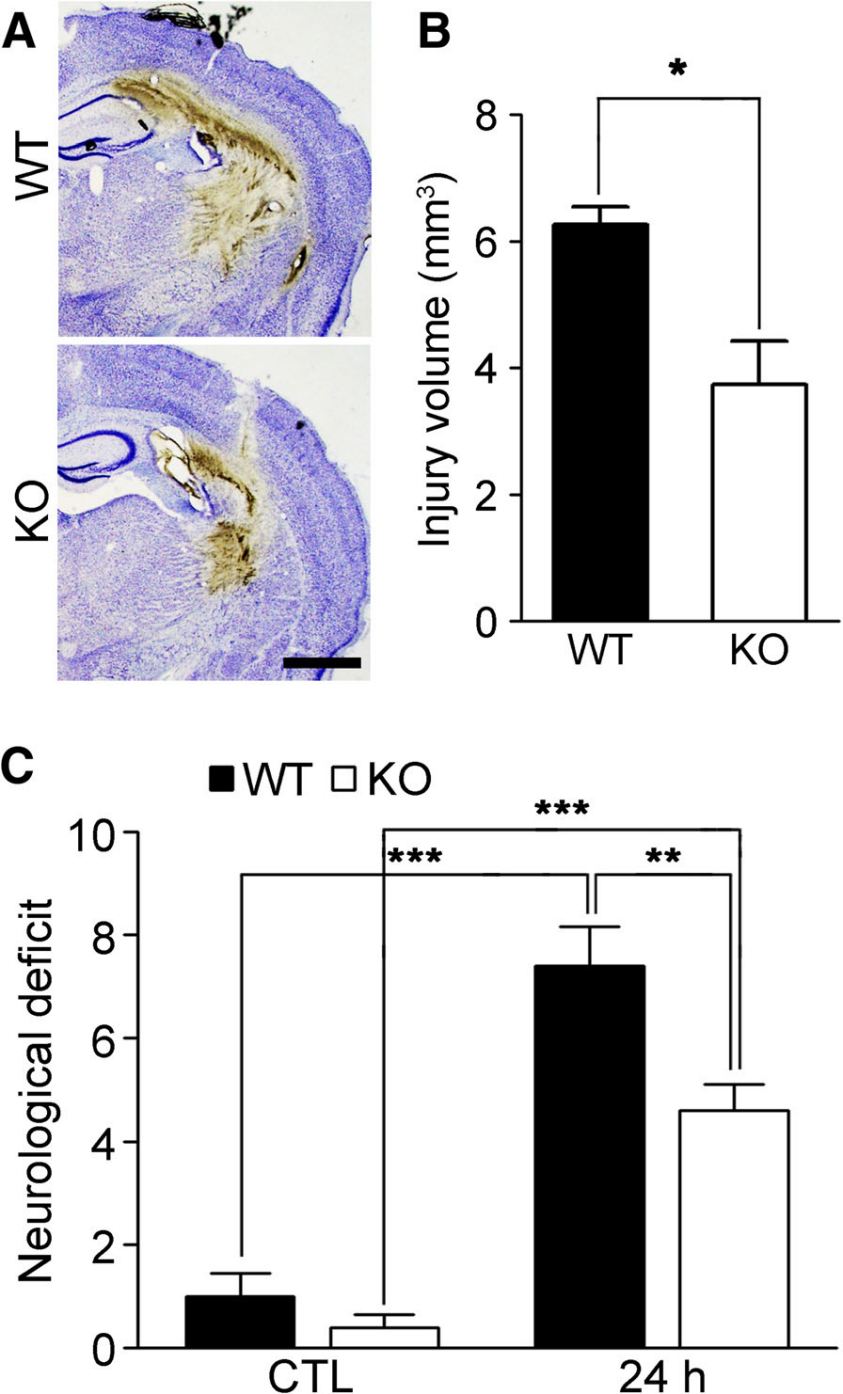
Hemin-induced ICH injury is attenuated in TLR2 KO mice. **a-b** Hemin-injected WT and TLR2 KO mouse brain sections were used for cresyl violet staining to measure brain damage. Scale bar: 1 mm. The injury volume (mm^3^) was calculated by multiplying the section thickness by the injured hemorrhagic area (* *p* < 0.05 vs. hemin-injected WT mice). **c** At 24 h after hemin administration, the neurological deficits were evaluated in the WT (*n* = 5) and TLR2 KO (*n* = 5) mice (** *p* < 0.01 vs. hemin-injected WT mice, *** *p* < 0.001 vs. control mice). For all graphs, the data are presented as mean ± SEM

Our previous study showed that TLR2 contributed to inflammatory responses and secondary brain damage after collagenase-induced ICH. We found that TLR2 expression was induced by hemin injection (Fig. 1f), suggesting a putative role for TLR2 in hemin-induced ICH injury. To determine if TLR2 was also involved in hemin-induced ICH, we introduced hemin into the striatum of TLR2 KO and WT mice. Histological assessment showed that the hemorrhagic area induced by hemin injection was significantly decreased in the TLR2 KO mice compared with WT mice (6.26 vs 3.73 mm^3^) (Fig. 2a, b). Likewise, the neurological deficit score 24 h after hemin injection was reduced in TLR2 mice (7.4 vs 4.6) (Fig. 2c).

**Fig. 3:**
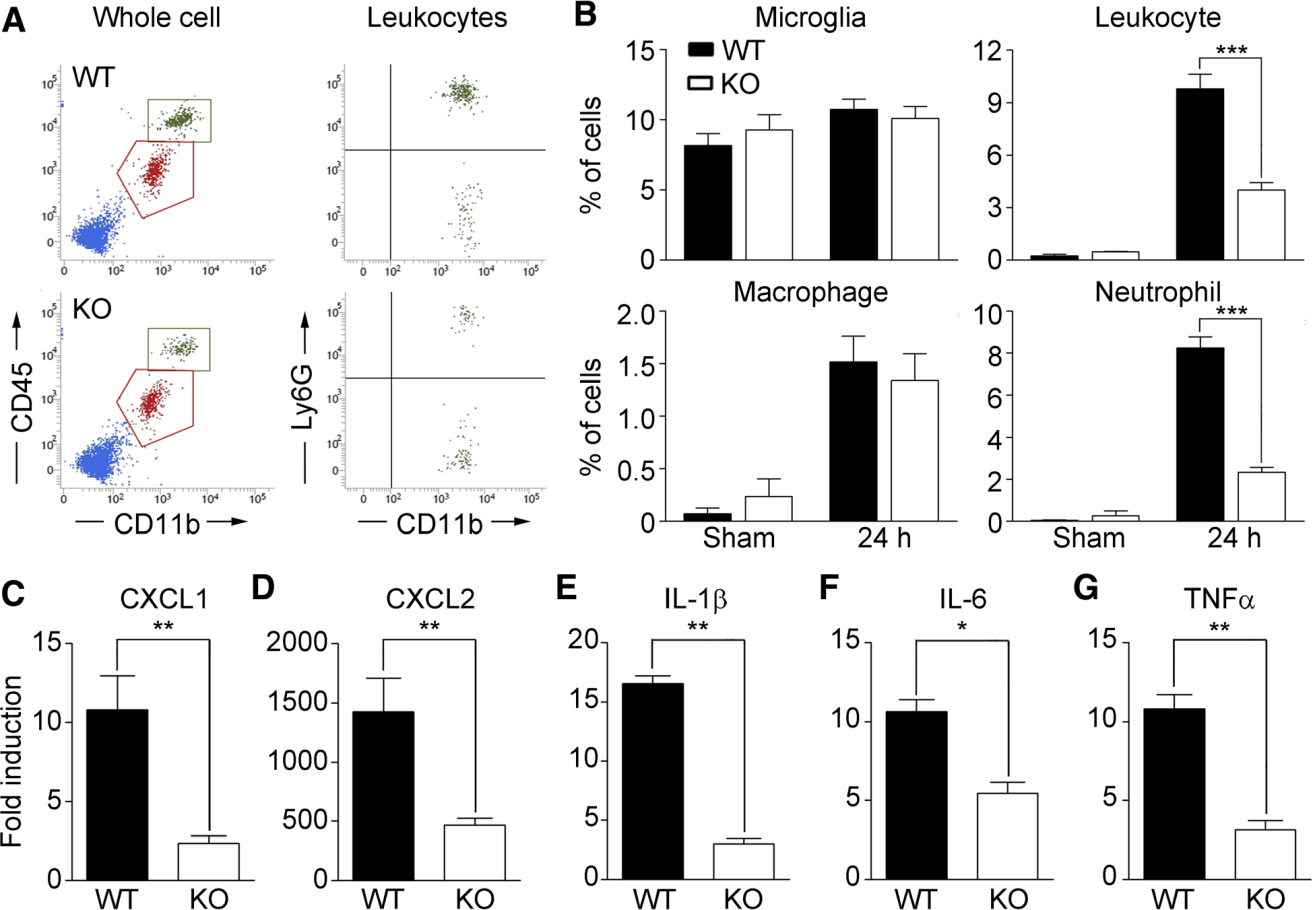
Hemin-induced inflammatory responses in the brain are compromised in TLR2 KO mice. **a-b** WT and TLR2 KO mice (*n* = 5 in each group) were administered 250 nmol of hemin in the striatum. After 24 h, the ICH-injured brains were dissociated into single-cell suspensions and the percentages of leukocytes (CD45^high^), macrophages (CD45^high^/CD11b^+^/Ly6G^−^), neutrophils (CD45^high^/CD11b^+^/Ly6G^+^), and microglia (CD45^med^/CD11b^+^) in the analyzed single cell population were determined using flow cytometry (*** *p* < 0.001 vs. hemin-injected WT mice). **c-g** Total RNA was isolated from the WT and TLR2 KO brain tissues (AP, 0.0 to −2.0 mm) 6 h after either saline- or hemin-administration, and used for quantitative real-time RT-PCR to measure CXCL1 (**c**), CXCL2 (**d**), IL-1β (**e**), IL-6 (**f**), and TNFα **g** mRNA levels. The mRNA levels of the hemin-injected mice were normalized to the levels of the saline-injected mice, and presented as fold induction (* *p* < 0.05, ** *p* < 0.01 vs. WT mice, *n* = 3). For all graphs, the data are presented as mean ± SEM

We next tested if hemin administration triggered inflammatory responses similar to those observed in collagenase-induced ICH. At 1 day after hemin administration, the CD45^med^/CD11b^+^ microglia population showed no significant change while the CD45^high^ blood-derived leukocyte population was increased 32-fold (from 0.3% to 9.7%) in the injured brain region (Fig. 3a and b). The Ly6G^−^/CD11b^+^ macrophage population and the Ly6G^+^/CD11b^+^ neutrophil population in the injured brain corresponded to 1.5% and 7.9% of the total cells, respectively (Fig. 3b). The increased percentage of macrophages in the TLR2 KO mice after hemin injection was comparable with that observed in WT mice. However, neutrophil infiltration into the injured brains was reduced more than 70% in the TLR2 KO mice, indicating that TLR2 expression is required for efficient neutrophil infiltration into injured brain tissue during hemin-induced ICH (Fig. 3b). To elucidate the mechanisms underlying neutrophil infiltration after hemin-induced ICH, we measured the mRNA expression levels of CXCL-1 and CXCL-2, two neutrophil-attracting chemokines, in the injured brain tissue after ICH. Following hemin administration, the transcript levels of CXCL-1 and CXCL-2 were increased 10.8-fold and 1424-fold, respectively, in WT mouse brain tissue. However, the CXCL-1 and CXCL-2 induction levels were decreased by 80% and 67%, respectively, in the TLR2 KO mice compared to the WT mice (Fig. 3c and d). Similarly, IL-1β, IL-6, and TNF-α expressions were upregulated 16.5-fold, 10.6-fold, and 10.8-fold, respectively, in WT perihematomal tissue, whereas the induction levels were decreased by 81%, 49%, and 77%, respectively, in the TLR2 KO mice (Fig. 3e-g). Taken together, these data indicate that the heme molecule induces neutrophil infiltration and proinflammatory gene expression in the brain parenchyma and that these effects require TLR2 expression.

**Fig. 4:**
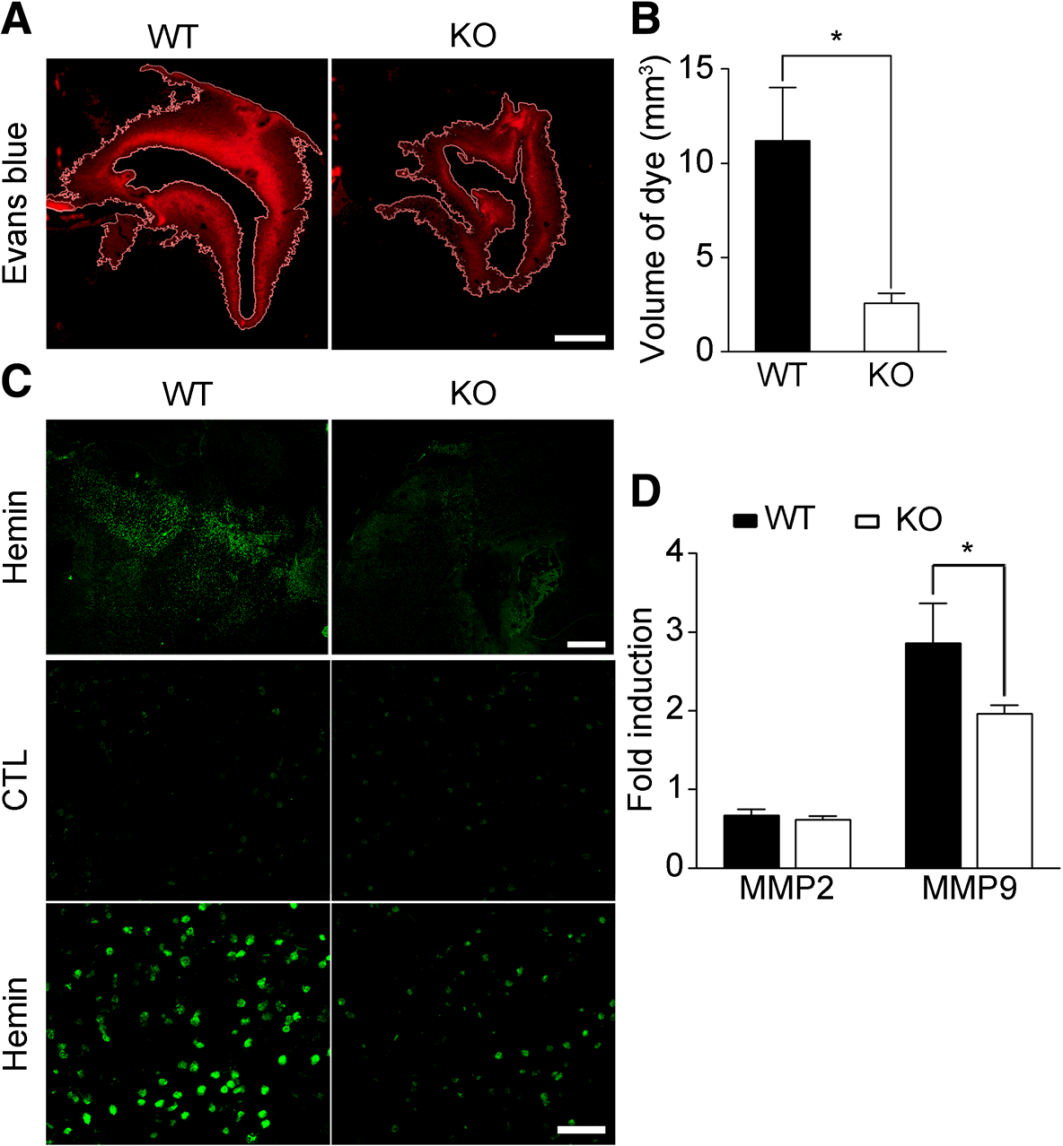
Hemin injection activates MMP9 and induces BBB damage via TLR2. **a-b** WT and TLR2 KO mice were administered 250 nmol of hemin in the striatum followed by intravenous* Evans blue* dye injection. After 24 h, brain sections were prepared and *Evans blue* staining was visualized to measure BBB damage. Scale bar: 1 mm. The injury volume (mm^3^) was calculated by multiplying the section thickness by the *Evans blue-stained* area. The data are expressed as mean ± SEM (* *p* < 0.05 vs. hemin-injected WT mice, *n* = 4). **c** The gelatinase activity in the injured brains of WT and TLR2 KO mice was measured using in situ zymography. The fluorescence due to gelatinase activity in the perihematomal region was visualized under a fluorescence microscope (upper four panels, scale bar: 500 μm). Magnified images are shown in the lower two panels (scale bar: 50 μm). **d** RNA was isolated from the injured tissue of WT and TLR2 KO mice 6 h after either saline- or hemin-injection (*n* = 4), and used for real-time RT-PCR to measure MMP2 and MMP9 mRNA levels. The mRNA levels of the hemin-injected mice were normalized to the levels of the saline-injected mice, and presented as fold induction. The data are presented as mean ± SEM (* *p* < 0.05 vs. WT mice)

To examine whether TLR2 is also involved in heme-induced BBB compromise, we performed Evans blue staining in WT and TLR2 KO mice. Upon intravenous Evans blue dye infusion at 24 h post-hemin injection, the dye was detected in a wide perihematomal region in brain sections from WT mice (Fig. 4a). In the TLR2 KO mice, however, the dye-stained area was significantly reduced (Fig. 4a). Quantification of the Evans blue-stained volume showed that it was reduced more than 70% in the TLR2 KO mice compared to WT mice (11.2 vs. 2.5 mm^3^) (Fig. 4b), once again confirming the pivotal role of TLR2 in heme-induced BBB compromise.

In our previous study, we showed that collagenase injection increased astrocyte MMP9 activity via TLR2 stimulation, which in turn compromised BBB integrity [6]. Similar to the collagenase-induced ICH model, hemin injection strongly increased gelatinase activity in the perihematomal region (Fig. 4c). Compared with WT mice, the heme-induced gelatinase activity was much lower in the TLR2 KO mice (Fig. 4c). Since both MMP2 and MMP9 have gelatinase activity, we measured the transcript levels of these genes in the perihematomal tissue. In WT mice, heme injection upregulated the MMP9 transcript by 2.9-fold, while the MMP2 transcript was slightly (0.7-fold) downregulated (Fig. 4d). In TLR2 KO mice, heme-induced MMP9 expression was reduced by 31% compared to the WT mice (Fig. 4d). These data suggest that TLR2 is required for maximal MMP9 expression and gelatinase activity in the perihematomal region after heme injection, which may lead to hemin-induced BBB damage.

**Fig. 5:**
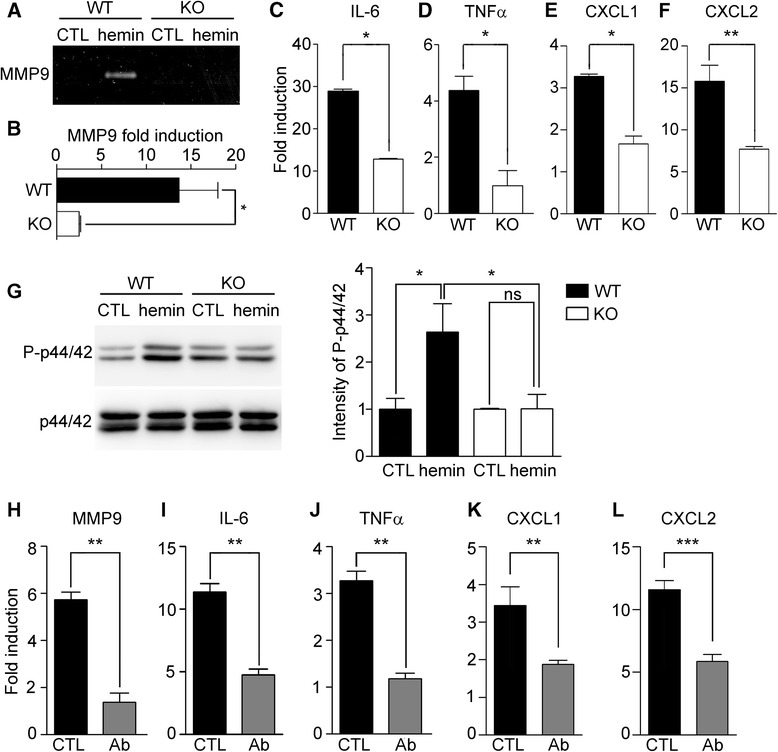
Hemin functions as an endogenous agonist of TLR2 to induce inflammatory astrocyte activation. Primary astrocytes were prepared from WT and TLR2 KO mice cerebra, and then stimulated with hemin (30 μM). **a** After 24 h, the cell culture supernatant from each sample was used for gel zymography to measure MMP9 activity. **b-f** Primary cultured astrocytes were treated with or without 30 μM of hemin for 6 h. Total RNA was prepared and used to measure MMP9 (**b**), IL-6 (**c**), TNFα (**d**), CXCL1 (**e**), and CXCL2 **f** transcript levels using real-time RT-PCR. The mRNA levels of the hemin-treated astrocytes were normalized to the levels of the astrocytes without hemin stimulation, and presented as fold induction (* *p* < 0.05, ** *p* < 0.01 vs. WT astrocytes, *n* = 3). **g** After 30 min of hemin stimulation, total protein extracts were prepared. Phosphorylated-p44/42 (P-p44/42) MAPK and total p44/42 MAPK were analyzed using Western blotting. Representative images are shown. The band intensities were quantified and are presented in the graph on the *right* (* *p* < 0.05, *n* = 3). **h-l**. Primary astrocytes were stimulated with hemin for 6 h, with or without a 1-h pretreatment with TLR2-neutralizing antibodies. Total RNA prepared from each sample was used to measure MMP9 (**h**), proinflammatory cytokine (**i**, **j**) and chemokine (**k**, **l**) transcript levels (**p < 0.01, ***p < 0.001, *n* = 3). For all graphs, the data are presented as mean ± SEM

In collagenase-induced ICH, MMP9 is activated in astrocytes, a major cell type in the BBB [10]. Therefore, we tested if the heme molecule directly induced MMP9 expression and/or stimulated MMP9 activity in astrocytes. Hemin treatment of primary cultured WT astrocytes stimulated MMP9 activity in the conditioned medium as measured by gel zymography (Fig. 5a). However, this heme-induced MMP9 activity was completely abolished in the conditioned medium of TLR2 KO astrocytes (Fig. 5a). Likewise, heme treatment induced MMP9 mRNA expression 13.7-fold in WT astrocytes compared to only 2.5-fold in TLR2 KO astrocytes (Fig. 5b). We also examined the induction of proinflammatory cytokines and chemokines after hemin treatment. Upon heme treatment, IL-6 and TNFα mRNA expression was upregulated 28.9-fold and 4.4-fold, respectively, in WT astrocytes; however, these induction levels were decreased by 56% and 77%, respectively, in the TLR2-deficient astrocytes (Fig. 5c, d). Similarly, CXCL1 and CXCL2 expression was upregulated 3.3-fold and 15.8-fold, respectively, in WT astrocytes compared to only 1.7-fold and 7.7-fold, respectively, in TLR2 KO astrocytes (Fig. 5e, f). We then tested the intracellular signaling pathways involved in hemin-induced inflammatory gene expression in astrocytes and found that hemin treatment induced p44/42 MAPK activation in WT astrocytes (Fig. 5g). This hemin-induced MAPK activation was also completely abolished in the TLR2-deficient astrocytes (Fig. 5g). Moreover, we confirmed that TLR2-neutralizing antibody treatment significantly decreased heme-induced MMP9, IL-6, and TNFα expression by 76%, 58%, and 64%, respectively (Fig. 5h-j). Neutrophil-attracting chemokine induction in WT and TLR2 KO astrocytes was also measured. The expression of CXCL1 and CXCL2 mRNA in the TLR2 KO astrocytes was decreased by 45% and 49%, respectively, compared to WT astrocytes (Fig. 5k, l). These data indicated that hememolecules directly interacted with astrocyte TLR2 and induced expression of inflammatory genes. Taken together, our data demonstrate that the heme molecule functions as an endogenous agonist of astrocyte TLR2, which stimulates MMP9 activity and induces proinflammatory cytokine and chemokine gene expression, thereby leading to secondary brain damage after ICH.

## Discussion

In this study, we showed for the first time that heme molecules released from hematomas function as endogenous agonists of astrocyte TLR2 and induce MMP9 activation, thereby leading to BBB damage and secondary brain injury after ICH. We previously reported that TLR2 was required for inflammatory responses, MMP9 activation, and BBB damage after collagenase-induced ICH, suggesting the involvement of putative damage-associated molecular patterns (DAMPs) that are released from the damaged brain tissue and activate astrocyte TLR2 [6]. However, the molecular identity of the endogenous TLR2 agonist had not been previously defined. In this study, we focused on the heme molecule as a candidate endogenous TLR2 agonist for two reasons. First, the tissue-damaging cytotoxic effects of heme molecules are well established. For instance, the free heme molecule is a potent inducer of proinflammatory cytokines in macrophages [11]. Also, administration of hemin into the brain has a tissue-damaging effect accompanied by inflammatory responses mimicking the pathological features observed during collagenase-induced ICH [8]. Second, DAMP molecules functioning as endogenous TLR agonists, such as heat shock proteins [12] and high-mobility group B1 [13], have features that are not usually detected by innate immune cells and are only exposed to the brain parenchyma upon tissue damage. We reasoned that heme molecules meet these criteria, since hemoglobin is the most abundant molecule in hematomas and is not exposed to the brain parenchyma under normal physiological conditions.

In support of this idea, a previous study implicated the heme molecule as a candidate DAMP molecule involved in secondary brain damage after ICH [8]. Based on in vivo work using TLR4 KO mice in which heme-induced brain damage and proinflammatory responses were severely reduced, the authors proposed that the heme molecule activated microglial TLR4 to trigger an inflammatory response [8]. However, the authors did not show a direct interaction of the heme molecule with TLR4. Instead, they suggested that heme may function as an endogenous agonist of microglial TLR4 in ICH. This hypothesis was based on a study by Figueiredo et al., who showed that heme molecules activated macrophages in a TLR4-dependent manner [11]. That study showed decreased heme-induced proinflammatory cytokine expression in TLR4 KO macrophages, but evidence of a direct interaction between heme and TLR4 was lacking. However, our study clearly demonstrated that heme activated MMP9 in astrocytes via TLR2, since the activation was abolished by TLR2-neutralizing antibodies. One possible explanation for this discrepancy is that heme may function as a DAMP for diverse TLR family members depending on the cell type, i.e., it may activate inflammatory gene expression in macrophages via TLR4, whereas it activates astrocyte MMP9 via TLR2. Alternatively, it is possible that the lack of heme effects in TLR4 KO macrophages is due to their reduced expression of TLR2. In support of this hypothesis, TLR2 expression has been reported to be severely compromised in TLR4 KO cells [14].

We also showed here for the first time that heme molecules can damage the BBB by inducing MMP9 activity in astrocytes. A previous study implicated heme molecules in inflammatory gene expression and brain tissue damage after ICH [8]. Correspondingly, we also showed that heme injection into the brain induced MMP9 activity and a compromised BBB, another hallmark of ICH. The critical role of aberrant MMP9 activation in BBB damage has been well documented [15, 16], and therefore, elucidating the MMP9 activation mechanism during ICH has been a key research goal in this field. Several studies have indicated that p44/42 MAPK activation was required for full induction of MMP9 transcription and activation [17, 18]. In our study, we found that hemin was able to activate p44/42 MAPK in a TLR2-dependent manner. Therefore, our data strongly support the possibility that heme molecules released from hematomas activate MMP9 via astrocyte TLR2-MAPK activation, which in turn leads to BBB compromise and subsequent secondary brain damage. Notably, it has been reported that HMGB1 released from damaged tissue may function as an endogenous agonist of TLR4 that aggravates ischemic brain damage in an ischemic stroke model [19]. One hypothesis is that the specific DAMP molecule responsible for the secondary brain damage depends on the stroke model. In ischemic stroke, which is associated with minimal bleeding, HMGB1 released from necrotic cells triggers inflammatory responses and leads to secondary brain damage. In hemorrhagic stroke, however, erythrocyte lysis in hematomas releases a large quantity of hemoglobin, which is derived from free heme molecules and is responsible for the subsequent inflammation.

In our study, we showed that hemin administration induced massive recruitment of immune cells, including macrophages and neutrophils, into injured brain parenchyma. Notably, neutrophil infiltration was attenuated in TLR2 KO mice, indicating that heme-TLR2 signaling contributed to neutrophil infiltration. ICH-induced neutrophil infiltration has been shown to contribute to brain tissue damage [20]. In contrast, infiltrated macrophages may promote wound healing and recovery from ICH injury in the brain [21]. Therefore, selective intervention in heme-TLR2 signaling has the potential to inhibit detrimental neutrophil infiltration while still maintaining beneficial macrophage infiltration after ICH. This strategy may be an efficient approach for regulating secondary brain damage after ICH.

## Conclusions

In this study, we revealed that the heme molecule can function as an endogenous agonist of astrocyte TLR2 to trigger inflammatory responses after ICH. Exogenous administration of hemin induced neutrophil infiltration, MMP9 activity in astrocytes, BBB leakage, neurological deficits, and secondary brain damage in WT mice. All of these ICH pathological features were reduced in TLR2 KO mice. In addition, heme-induced MMP9 activation and proinflammatory cytokine expression in primary astrocytes were reduced in TLR2 KO astrocytes. Finally, heme-induced astrocyte activation was blocked by TLR2-neutralizing antibodies. Taken together, our data show that heme released into the brain parenchyma after ICH activates astrocyte TLR2 and induces an inflammatory response that contributes to secondary brain damage after ICH.

## Materials and methods

### Animals

TLR2 KO mice [22] were generously provided by Dr. S. Akira (Department of Host Defense, Osaka University, Osaka, Japan). The TLR2 KO mice had been backcrossed to the C57BL/6 background for more than 10 generations; C57BL/6 mice purchased from Daehan Bio Link (Eumsung, Korea) were used as WT control mice. The mice were housed at 23 ± 2 °C with a 12-h light-dark cycle and provided food and water ad libitum. All surgical and experimental procedures were reviewed and approved by the Institutional Animal Care and Use Committee (IACUC) at Seoul National University.

### ICH model and hemorrhagic injury volume analysis

WT and TLR KO mice (8- to 10*-*week-old males, 22–25 g) were anesthetized and placed on a stereotaxic apparatus (myNeuroLab, St. Louis, MO, USA). The animals were injected with saline or hemin (250 nmol in 2.6 μl PBS; Sigma, St. Louis, MO, USA) at a rate of 0.4 μl/min. After 5 min, the needle was removed in three intermediate steps over 3 min to minimize backflow*.* The incision was cleaned with saline and sutured, after which the animals were kept on a warm pad during recovery. To prepare brain tissue sections, the animals were deeply anesthetized and perfused with saline, and then the brain tissue was harvested and fixed with 4% paraformaldehyde. The brains were then quickly frozen and cut into serial coronal sections (50-μm thickness) using a cryostat (CM3050S, Leica Biosystems, Nussloch, Germany). The sections were collected as free-floating sections in cold PBS and then used for histochemical analysis. Brain slices from different levels of the injured hemorrhagic area were selected from each mouse brain and used for cresyl violet staining. The injured area was quantified using Image-Pro Plus software (Media Cybernetics, Inc., Rockville, MD, USA), and the injury volume was calculated in cubic millimeters (mm^3^) by multiplying the section thickness by the measured injury areas as described elsewhere [23].

### Evaluation of neurological deficits

Neurological deficits were assessed at 24 h following hemin injection. An experimenter blinded to the mouse genotypes scored all mice for neurological deficits using a 28-point neurological scoring system [9]. The tests included body symmetry, gait, climbing, circling behavior, front limb symmetry, and compulsory circling. Each test was graded from 0 to 4, yielding a maximum deficit score of 28. The mice were sacrificed for further analysis immediately following the testing.

### Determination of BBB permeability

To evaluate BBB permeability, mice were administered Evans blue dye (2% in saline, 4 ml per kg) by intravenous injection 2 h after hemin injection. After 24 h, the brain tissue was harvested, fixed with 4% paraformaldehyde, and placed in 30% sucrose in PBS for 48 h at 4 °C. The brains were then quickly frozen and cut into serial coronal sections (50-μm thickness) using a cryostat. A single section was collected every 8 consecutive sections, mounted on a slide, and then visualized under a light microscope. The Evans blue-stained area in each section was quantified using Image-Pro Plus software and summed throughout the injured hemorrhagic brain area. Total injury volume was calculated in cubic millimeters (mm^3^) by multiplying the section thickness by the measured area.

### Primary glial cell culture

Primary mixed glial cultures were prepared as previously described [24]. Briefly, mixed glial cultures were prepared from postnatal day 1 WT or TLR2 KO mice. After removing the meninges from the cerebral hemisphere, the tissue was dissociated into a single-cell suspension by gentle repetitive pipetting. The cells were cultured in DMEM supplemented with 10 mM HEPES, 10% FBS, 2 mM L-glutamine, and 1X antibiotic/antimycotic in 75 cm^2^ flasks at 37 °C in a 5% CO_2_ incubator. The medium was changed every 5 days. After 2 weeks, the microglia were removed by treating the cells with 100 mM L-leucine methyl ester for 60 min, harvested using trypsinization (0.25% trypsin, 0.02% EDTA), and seeded in 6-well dishes. To block hemin-induced TLR2 activation, the cells were preincubated with 50 μg/ml of TLR2 antibody (Biolegend, San Jose, CA, USA).

### Real-time RT-PCR

Real-time RT-PCR was performed using SYBR Green PCR Master Mix (ABI, Warrington, UK) as described previously [25]. The reactions were performed in duplicate in a total volume of 10 μl containing 10 pM primer, 4 μl cDNA, and 5 μl SYBR Green PCR Master Mix. The mRNA levels of each target gene were normalized to that of GAPDH mRNA. Fold-induction was calculated using the 2^-∆∆CT^ method, as previously described [26]. All real-time RT-PCR experiments were performed at least three times and are presented as the mean ± SEM unless otherwise noted. The following primers were used for real-time RT-PCR: TLR2 forward: 5′-CCT AGA AGT GGA AAA GAT GTC GTT CA-3′; TLR2 reverse: 5′-GAA GAA AAC GGA ATT CTC TTT TCG AC-3′; CXCL1 forward: 5′-CCG AAG TCA TAG CCA CAC TCA A-3′; CXCL1 reverse: 5′-GCA GTC TGT CTT TCT CCG TTA C-3′; CXCL2 forward: 5′-AGA CAG AAG TCA TAG CCA CTC TCA AG-3′; CXCL2 reverse: 5′-CCT CCT TTC CAG GTC AGT TAG C-3′; IL-1β forward: 5′-TTG TGG CTG TGG AGA AGC TGT-3′; IL-1β reverse: 5′-AAC GTC ACA CAC CAG GTT-3′; IL-6 forward: 5′- TCC ATC CAG TTG CCT TCT TGG-3′; IL-6 reverse: 5′-CCA CGA TTT CCC AGA GAA CAT G-3′; TNF-α forward: 5′-AGC AAA CCA AGT GGA GGA-3′; TNF-α reverse: 5′-GCT GGC ACC ACT AGT TGG TTG T-3′; MMP-9 forward: 5′-CAT TCG CGT GGA TAA GGA GT-3′; MMP-9 reverse: 5′-ACC TGG TTC ACC TCA TGG TC-3′; GAPDH forward: 5′-CAC CCT GTT GCT GTA GCC GTA T-3′; GAPDH reverse: 5′-AGG TCA TCC CAG AGC TGA ACG-3′.

### In situ zymography

The in situ gelatinolytic activity was measured in frozen sections (16-μm thickness) as described previously [27]. At 24 h following ICH induction, the brains were removed and immediately frozen on dry ice. Fresh sections were incubated with fluorescein-conjugated DQ gelatin substrate (Invitrogen, Carlsbad, CA, USA) for 2 h and subsequently fixed and mounted with VectaShield (Vector Laboratories, Burlingame, CA, USA) medium. In this assay, cleavage of DQ gelatin by MMPs results in green fluorescent products. The images were then captured using a confocal laser scanning microscope (LSM700, Carl Zeiss, Germany).

### Gel zymography

Glial cells were incubated with or without hemin in serum-free medium at 37 °C in a 5% CO_2_ incubator for 24 h, after which the supernatants were collected. To determine the protein concentration, 3 volumes of ethanol were added to each supernatant, after which the mixture was incubated at −20 °C for 1 h then centrifuged at 13,000 rpm for 30 min at 4 °C. The resultant precipitates were resuspended in sample buffer (0.25 M Tris pH 6.8, 10% glycerol, 2% SDS) and subjected to SDS-PAGE in 8% polyacrylamide gels containing 1 mg/ml gelatin. After electrophoresis, the gels were incubated at RT in 2.5% Triton X-100 for 30 min and then incubated with zymogram developing buffer for 30 h at 37 °C. The gels were stained with 0.25% Coomassie Brilliant Blue and destained to visualize MMP9 activity.

### Flow cytometry

To measure inflammatory-cell infiltration in the brain, flow cytometry was performed. Single-cell suspensions were prepared from each mouse brain, washed with 2% fetal bovine serum in PBS, and incubated with Fc block™ (BD Biosciences, San Jose, CA, USA) for 10 min at 4 °C. After washing twice with 2% FBS in PBS, the cells were incubated with PE-conjugated anti-CD45, FITC-conjugated anti-CD11b, and APC-conjugated anti-Ly6G antibodies (BD Biosciences) for 30 min at 4 °C. A BD FACSVerse™ flow cytometer (BD Biosciences) was used to measure the microglia (CD45^med^/CD11b^+^/Ly6G^−^), leukocyte (CD45^high^), macrophage (CD45^high^/CD11b^+^/Ly6G^−^), and neutrophil (CD45^high^/CD11b^+^/Ly6G^+^) populations as defined elsewhere [28, 29]. The data were collected and analyzed using BD FACSuite™ software (BD Biosciences).

### Western blot assay

For Western blots, the protein samples were separated by SDS-PAGE on 12% gels then transferred to nitrocellulose membranes. After blocking the nonspecific binding sites with 3% BSA in TBST (20 mM Tris pH 7.4, 0.1% Tween 20, 150 mM NaCl), the membranes were incubated with rabbit anti-phospho-p44/42 (1:1000 dilution; Cell Signaling, Danvers, MA, USA), or rabbit anti-p44/42 (1:1000 dilution; Cell Signaling) antibodies. The proteins were detected with horseradish peroxidase-conjugated secondary antibodies using the West Save Gold western blot detection kit (Ab Frontier, Seoul, Korea). The immunoreactive bands were visualized using a Chemiluminescence imaging system (Syngene, Cambridge, United Kingdom).

### Statistical analysis

Differences between the WT and TLR2 KO mice were determined using the Student’s *t*-test or one-way ANOVA. All data are presented as the mean ± SEM; differences were considered significant when the *p-*value was less than 0.05.

## Abbreviations

BBB: Blood-brain barrier
CD: Cluster of differentiation
DAMP: Damage associated molecular patterns
ICH: Intracerebral hemorrhage
IL: Interleukin
KO: Knock-out
MMP: Metalloproteinase
TLR: Toll-like receptor
TNF: Tumor necrosis factor
WT: Wild type
